# Molecular Docking Studies of Marine Diterpenes as Inhibitors of Wild-Type and Mutants HIV-1 Reverse Transcriptase

**DOI:** 10.3390/md11114127

**Published:** 2013-10-29

**Authors:** Leonardo A. Miceli, Valéria L. Teixeira, Helena C. Castro, Carlos R. Rodrigues, Juliana F. R. Mello, Magaly G. Albuquerque, Lucio M. Cabral, Monique A. de Brito, Alessandra M. T. de Souza

**Affiliations:** 1 Laboratory of Antibiotics, Biochemistry, Education and Molecular Modeling (LABiEMol), Institute of Biology, Fluminense Federal University (UFF), Campus of Valonguinho, Niteroi, 24210-130, RJ, Brazil; E-Mail: leossj@hotmail.com; 2 Laboratory of Natural Products of Marine Algae, Institute of Biology, Fluminense Federal University (UFF), Campus of Valonguinho, Niteroi, 24210-130, RJ, Brazil; E-Mail: valerialaneuville@gmail.com; 3 Laboratory of Molecular Modeling and QSAR (ModMolQSAR), Center for Health Sciences, Federal University of Rio de Janeiro (UFRJ), Faculty of Pharmacy, 21941-590, Rio de Janeiro, RJ, Brazil; E-Mails: rangelfarmacia@gmail.com (C.R.R.); foncor@gmail.com (J.F.R.M.); 4 Laboratory of Molecular Modeling (LabMMol), Chemistry Institute, Federal University of Rio de Janeiro (UFRJ), 21949-900, Rio de Janeiro, RJ, Brazil; E-Mail: magaly@iq.ufrj.br; 5 Laboratory of Industrial Pharmaceutical Technology (LabTIF), Faculty of Pharmacy, Center for Health Sciences, Federal University of Rio de Janeiro (UFRJ), 21941-590, Rio de Janeiro, RJ, Brazil; E-Mail: lmcabral@pharma.ufrj.br; 6 Laboratory of Computational Medicinal Chemistry, Faculty of Pharmacy, Fluminense Federal University (UFF), 24241-000, Niteroi, RJ, Brazil; E-Mail: moniquebrito@id.uff.br

**Keywords:** HIV-1, mutants, molecular docking, brown algae diterpenes, antiviral, reverse transcriptase

## Abstract

AIDS is a pandemic responsible for more than 35 million deaths. The emergence of resistant mutations due to drug use is the biggest cause of treatment failure. Marine organisms are sources of different molecules, some of which offer promising HIV-1 reverse transcriptase (RT) inhibitory activity, such as the diterpenes dolabelladienotriol (THD, IC_50_ = 16.5 µM), (6*R*)-6-hydroxydichotoma-3,14-diene-1,17-dial (HDD, IC_50_ = 10 µM) and (6*R*)-6-acetoxydichotoma-3,14-diene-1,17-dial (ADD, IC_50_ = 35 µM), isolated from a brown algae of the genus *Dictyota*, showing low toxicity. In this work, we evaluated the structure-activity relationship (SAR) of THD, HDD and ADD as anti HIV-1 RT, using a molecular modeling approach. The analyses of stereoelectronic parameters revealed a direct relationship between activity and HOMO (Highest Occupied Molecular Orbital)-LUMO (Lowest Unoccupied Molecular Orbital) gap (E_LUMO_–E_HOMO_), where antiviral profile increases with larger HOMO-LUMO gap values. We also performed molecular docking studies of THD into HIV-1 RT wild-type and 12 different mutants, which showed a seahorse conformation, hydrophobic interactions and hydrogen bonds with important residues of the binding pocket. Based on *in vitro* experiments and docking studies, we demonstrated that mutations have little influence in positioning and interactions of THD. Following a rational drug design, we suggest a modification of THD to improve its biological activity.

## 1. Introduction

Acquired immunodeficiency syndrome (AIDS) was first identified in 1981 and, since then, 26 million people have died of HIV-1 related causes worldwide [1]. Nowadays, HIV-1 infection has continued to increase, with an estimated 33.2 million individuals living with the virus [2].

HIV-1 reverse transcriptase (RT) is the major target for antiretroviral therapy since it plays a key role in the virus replication cycle, converting the single-stranded viral RNA genome into double-stranded DNA [3]. Structurally, this enzyme is an heterodimer composed of p66 and p51 subunits [4,5].

Non-nucleoside RT inhibitors (NNRTIs) are structurally diverse noncompetitive inhibitors that bind at a hydrophobic pocket located 10 Å from the catalytic site [6]. Nowadays, there are approximately 30 classes of structurally unrelated NNRTIs. Among them, etravirine is the newest NNRTI approved by the U.S. Food and Drug Administration (FDA) for treatment of HIV-1 infected patients currently resistant to NNRTI-based therapies. The major limitation of NNRTIs in clinical use is the HIV-1 RT mutations emergence that blocks the binding of the inhibitor [7]. Single mutations such as Leu100Ile, Lys101Glu, Lys103Asn, Val106Ala, Val108Ile, Tyr181Cys, Tyr188Leu, Gly190Ala, Pro225His, Phe227Leu and double mutations as Lys103Asn/Tyr181Cys, Lys103Asn/Val108Ile, Lys103Asn/Pro225His, Lys103Asn/Leu100Ile have been described in the literature as a result of prolonged use of NNRTIs [8,9,10,11,12,13,14,15,16,17]. Thus, the search for new therapeutic agents that are able to act against both this enzyme wild type and mutants is urgently needed.

The first evidence of seaweed metabolites’ antiviral properties have been demonstrated over 50 years ago [18]. However, it is only since 1970 that several research groups worldwide started systematic screening of extracts of algae to find products with biological activity and, in particular, an antiviral profile [19]. A number of marine natural products have been demonstrated to present pharmacological activities against a wide range of pathogens, including HIV-1 [20,21].

**Figure 1:**
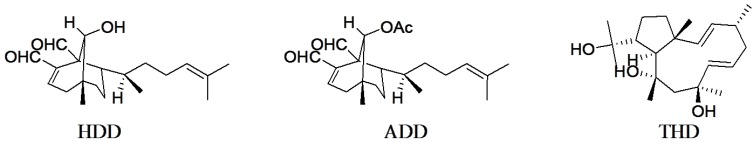
Chemical structures of HDD, ADD and THD isolated from *Dictyota* species.

Literature describes diterpenes isolated from the *Dictyota* brown algae species with potential antiviral activity [22]. Previously, our group showed that the main diterpene compounds isolated from *Dictyota menstrualis* (Hoyt) Schnetter, Hörning & Weber-Peukert, identified as (6*R*)-6-hydroxydichotoma-3,14-diene-1,17-dial (HDD, IC_50_ = 10 µM) and its acetate derivative (6*R*)-6-acetoxydichotoma-3,14-diene-1,17-dial (ADD, IC_50_ = 35 µM) (Figure 1), exhibit inhibitory activities against HIV-1 RT in a dose-dependent form [23]. A similar antiviral profile was observed in a dollabelane diterpene isolated from *Dictyota pfaffii* Schnetter, (1R*,2*E*,4R*,6*E*,10S*,11S*,12R*)-8,10,18-trihydroxy-2,6-dolabelladiene (THD, IC_50_ = 16.5 µM) (Figure 1). Additional studies revealed that THD acts as a NNRTI (*K*_i_ = 7.2 µM) when testing against a large panel of HIV-1 strains isolated harboring NNRTI resistance-associated mutations with no cross-resistance with clinically available NNRTIs [24,25,26]. In addition, studies showed that THD has low toxicity in the administered dose range.

In pursuit of this goal, herein we evaluated these diterpenes’ structure-activity relationship (SAR) and the HIV-1 RT inhibitory activity, by calculating most fundamental molecular descriptors, since these molecules are already known to have inhibitory effects on the HIV-1 RT enzyme. Furthermore, the analysis of the binding mode of THD with the non-nucleoside inhibitor binding pocket (NNIBP) of RT wild-type and mutants was performed using a molecular docking approach.

## 2. Results and Discussion

### 2.1. Structure-Activity Relationship and ADMET Evaluation of Diterpenes

The overall analysis of the calculated molecular descriptors showed that, despite the different chemical structures, nevirapine (a NNRTI) and diterpenes have similar values, probably with no direct correlation with antiviral activity. However, the HOMO-LUMO gap values (E_LUMO_-E_HOMO_) of the most active diterpene (THD) are much higher than the values of HDD, ADD and nevirapine (Table 1). This data suggest us that the HOMO-LUMO gap value may be important to antiviral profile since it is directly related to the stability of a molecule in which high HOMO-LUMO gap values indicates high internal stability. The molecular dipole moment (µ) was lower for the most potent diterpene (1.73 D) than in the least potent ones (4.40 and 5.28 D) (Table 1). Since the NNRTI binding pocket is essentially hydrophobic, we can infer that the less polarized the molecule is, the better its activity. Also, the size of these molecules seems to be important for activity, since the binding pocket is known for being limited in both size and elasticity to accommodate molecules [27]. It is important to notice that these results are an initial step to understand and generate a proper antiviral profile to this class of marine natural products with small and complex structures.

**Table 1:** Comparison of *in vitro* antiviral activity (EC_50_), HIV-1 RT inhibitory activity (IC_50_), citoxocicity (CC_50_), selectivity index (SI), and theoretical parameters: molecular dipole moment (μ), HOMO (Highest Occupied Molecular Orbital) and LUMO (Lowest Unoccupied Molecular Orbital) energies and gaps, cLogP, molecular mass (MM), hydrogen bond acceptor (HBA), and hydrogen bond donor (HBD) of nevirapine (NVP) and diterpenes HDD, ADD, THD.

#	EC_50_ (µM) ^a^	IC_50_ (µM) ^b^	CC_50_ (µM) ^a^	SI ^c^	µ (D)	E_HOMO_ (eV)	E_LUMO_ (eV)	_HOMO-LUMO_ gap (eV) ^d^	Lipinski’ Rule-of-5			
									cLogP	MM (Da)	HBA	HBD
NVP	0.4	0.5	>100	>250	2.67	−8.50	2.67	11.17	2.91	260	5	1
HDD	40.0	10.0	>200	>5	4.40	−8.90	2.51	11.41	3.95	318	3	1
ADD	70.0	35.0	>200	>2.86	5.28	−8.83	2.60	11.43	4.44	360	3	0
THD	8.4	16.5	500	59.5	1.73	−8.84	4.31	13.15	3.59	322	3	3

^a^ [25]; ^b^ [23]; ^c^ SI = CC_50_/EC_50_; ^d^ HOMO-LUMO gap = E_LUMO_ − E_HOMO_.

As these diterpenes may be considered for oral delivery, they were submitted to the analysis of the Lipinski “Rule-of-5” parameters. This rule determines if a chemical compound presents absorption and permeation across membranes’ properties that would make it a likely orally active drug in humans. According to Lipinski, molecules violating more than one of these rules may have problems with bioavailability [28]. Ours results showed that all diterpenes fulfilled the Lipinski’ Rule-of-5 (number of hydrogen bond acceptors, HBA ≤ 10 and donors, HBD ≤ 5; octanol-water partition coefficient, LogP ≤ 5; molecular mass, MM ≤ 500 Da) despite their chemical structural differences from the antivirals studied herein (Table 1).

**Figure 2:**
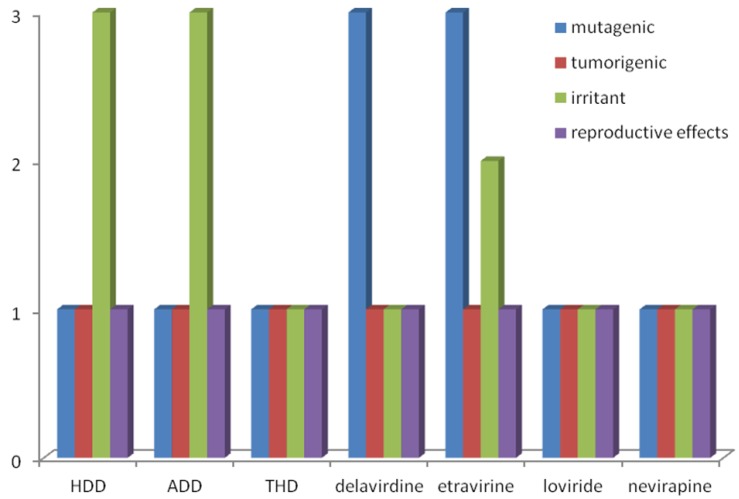
Theoretical toxicological profile of diterpenes HDD, ADD and THD and antiviral drugs delavirdine, etravirine, loviride and nevirapine.

The three diterpenes were also submitted to an *in silico* toxicity screening [29] to analyze their predicted toxicity risks (mutagenic, irritant, tumorigenic, and reproductive effects) (Figure 2). All theoretical toxicity evaluations of THD were better than those observed for delavirdine and etravirine, the antivirals currently used in HIV/AIDS therapy, presenting high mutagenic effects (Figure 2). These data reinforced the potential profile of these diterpenes but it is important to note that the toxicity predicted herein neither is a fully reliable toxicity prediction, nor guarantees that these compounds are completely free of any toxic effect. However, these theoretical results help to strengthen the promising profile of these diterpenes that has previously been inferred by experimental cytotoxicity assay (Table 1) [25].

### 2.2. Molecular Docking

#### 2.2.1. Validation of the Docking Performance and Accuracy

The validation of the docking accuracy was performed by docking the native co-crystallized TIBO ligand into an RT binding site. The comparison of re-docking results with the co-crystallized form showed success rates with the docked ligand strictly superimposed with the crystallized conformation with RMSD = 0.55 Å indicating that the used scoring function is successful. These values were small enough and supported the hypothesis that experimental binding modes could be reproduced with accuracy using this protocol.

#### 2.2.2. Molecular Docking with HIV-1 RT Wild Type

**Figure 3:**
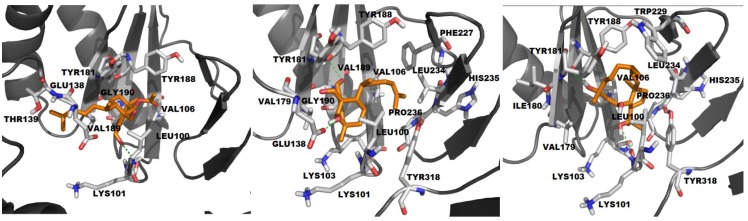
Comparison of the docking complexes of ADD, HDD and THD with HIV-1 RT wild-type, respectively, including the parameters binding energy (BE, kcal/mol), number of interactions (NI), clusters (NC), and conformations on lowest energy cluster (CE) van der Waals (vdW) and hydrogen bonding (H-bond).

*HIV-1 RT wild type and HDD:* The complex of HIV-1 RT wild-type and HDD revealed van der Waals interactions between the diterpene and residues Leu100, Lys101, Lys103, Val106, Val179, Tyr181, Val189, Gly190, Phe227, Leu234, His235, Pro236 and Tyr318, as well as hydrogen bonds with Tyr188 backbone (d = 2.73 Å) and Glu138 side chain (d = 1.97 Å) of p66 and p51 subunit, respectively (Figure 3). Despite the conformational similarity between HDD and classic NNRTIs, we detected the absence of some of the butterfly-like characteristics, such as hydrogen bond donors and acceptors groups in the vicinity of the Lys101 and hydrophobic domain in the vicinity to the hydrophobic residues (Tyr188, Tyr181 and Trp229), possibly related to HDD inhibitory activity lower than the THD and nevirapine.

*HIV-1 RT wild type and ADD:* The best pose of ADD showed van der Waals interactions with RT residues Leu100, Val106, Val179, Tyr181, Tyr188, Val189 and Gly190, a hydrogen bond with Lys101 backbone (d = 2.21 Å) of its p66 subunit and van der Waals interactions with residues Glu138 and Thr139 of p51 subunit (Figure 3). ADD interacts out of the limits of NNIBP, where a significant part of the inhibitor is beyond the hydrophobic pocket, as delimited in the β-sheet (β6-β9-β10 and β12-β13-β14) by residues Tyr181, Tyr188 and Trp229 and above the entrance of the pocket (Pro95, Leu100, Lys101 and Lys103). This interaction mode is similar to delavirdine, which extends into the pocket, held by a hydrogen bond with residues in NNIBP. Herein, we can compare HDD and ADD to first generation NNRTIs, due to their similar conformation with TIBO and delavirdine, respectively [24].

*HIV-1 RT wild type and THD:* THD docking showed all 50 conformations in a single cluster with a different conformation from other diterpenes where the butterfly-like conformation was not observed. Two hydrogen bonds were observed with Tyr188 backbone (d = 3.06 Å and 2.70 Å, respectively) of the enzyme in addition to the van der Waals interactions with Leu100, Lys103, Val106, Val179, Ile180, Tyr181, Trp229, Leu234, His235, Pro236 and Tyr318 residues and hydrogen bond with Lys101 backbone (Figure 3). Generally, the orientation of an NNRTI at the hydrophobic pocket is stabilized by a hydrogen bonding with Lys101 as well as π–π stacking interactions with the aromatic side chains of Tyr181 and Tyr188 residues [30]. As diterpenes have no aromatic substituents to support π–π interactions, the hydrogen bond with Tyr188 seems to be important for the correct orientation and stabilization of this molecule at the RT binding pocket. In addition, a seahorse conformation [12] was observed in the complex THD/HIV-1 RT. This is a unique conformation that allows the molecule to perform van der Waals interactions with a larger number of residues including Trp229, a highly conserved residue of the binding site.

**Table:** 

#	BE	NI	NC	CE	vdW	H-bond
**ADD**	−3.88	10	5	23, 21, 2, 3, 1	L100, V106, V179, Y181, Y188, V189, G190, E138, T139	K101 (2.21)
**HDD**	−4.96	16	5	13, 11, 17, 8, 1	L100, K101, K103, V106, V179, Y181, Y188, V189, G190, F227, L234, H235, P236, Y318	Y188 (2.73) E138 (1.97)
**THD**	−6.3	13	1	50	L100, K103, V106, V179, I180, Y181, W229, L234, H235, P236, Y318	K101 (3.06) Y188 (2.70)

#### 2.2.3. Diterpenes and HIV-1 RT Mutants Complexes Evaluation

**Figure 4:**
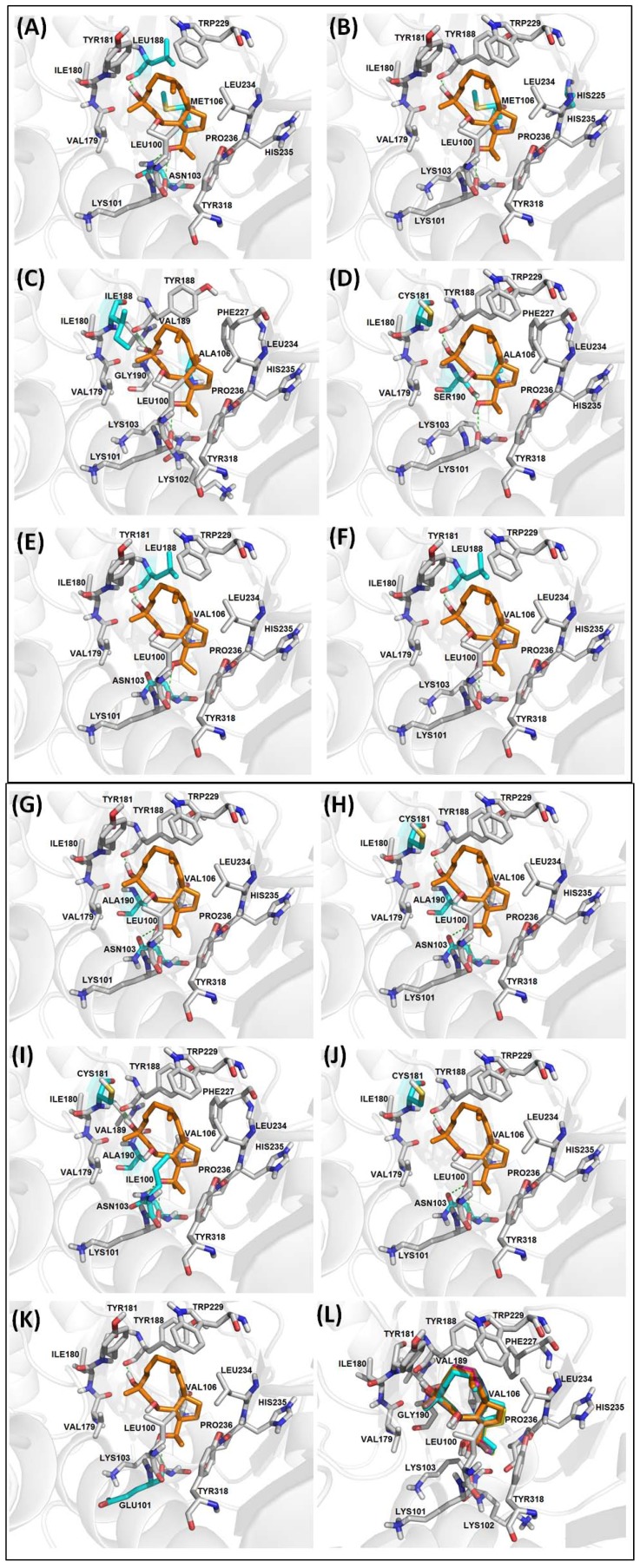
Molecular docking of THD on NNIBP of HIV-1 RT mutants (**A**) RT-1, (**B**) RT-2, (**C**) RT-3, (**D**) RT-4, (**E**) RT-5, (**F**) RT-7, (**G**) RT-8, (**H**) RT-9, (**I**) RT-10, (**J**) RT-11, (**K**) RT-12, (**L**) Superposition of all complexes. Ligand is shown in orange, residues involved on the interactions between THD and the enzyme are shown in gray, mutated residues are highlighted in cyan and hydrogen bonds in green.

Previous *in vitro* studies from our group showed that THD is capable to inhibit drug-resistant HIV-1 RT mutants [26]. Our molecular docking analysis of the complexes, constructed using 12 mutants and THD, showed that the best result converged predominantly to the most populous and lowest energy cluster, suggesting a more stable and preferred conformation (Figure 3). An overall analysis of the best docking results with RT mutants revealed that THD preserves the interactions with the same residues. However, for some mutations, new van der Waals interactions were observed, such as Lys102, Val189, Gly190 and Phe227 in RT-3; Phe227 in RT-4; Ala190 in RT-8; Ala190 in RT-9; and Val189, Ala190, and Phe227 in RT-10 and hydrogen bonds with Lys103 in TR-1, RT-8, RT-9, RT-10, RT-11, and RT-12; Ser190 in TR4 (Figure 4 and Table 2).

Superposition of all structures of the THD in complex with HIV-1 RT mutants showed the ligand in the hydrophobic pocket and a slight displacement that occurs according to the mutation in the vicinity (Figure 4).

Resistance mutations associated with NNRTI treatment failure occur primarily in and around the NNIBP [12]. Nevertheless, these mutations apparently do not seem to interfere with THD inhibitory activity. With the exception of RT-6, THD interacts with all mutants studied through van der Waals interactions with Val179, Ile180, Leu234, His235, Pro236 and Tyr318 residues and hydrogen bonds with Lys/Glu101 (distances ranging from 2.67 to 3.10 Å), and Tyr/Leu188 (distances ranging from 2.57 to 2.80 Å).

**Table 2:** Comparison the parameters of the docking complexes of THD /HIV-1 RT wild-type with the mutants and including binding energy (BE, kcal/mol), number of interactions (NI), clusters (NC) and conformations on lowest energy cluster (CE), van der Waals (vdW) and hydrogen bonding (H-bond) interactions.

RT	Mutation	BE	NI	NC	CE	vdW	H-bond
WT	None	−6.30	13	1	50	L100, K103, V106, V179, I180, Y181, W229, L234, H235, P236, Y318	K101 (3.06), Y188 (2.70)
RT-1	K103N, V106M, Y188L	−6.03	13	2	49, 1	L100, M106, V179, I180, Y181, W229, L234, H235, P236, Y318	K101 (3.14), N103 (2.77), L188 (2.76)
RT-2	V106M, P225H	−6.14	13	1	50	L100, K103, M106, V179, I180, Y181, W229, L234, H235, P236, Y318	K101 (3.08), Y188 (2.75)
RT-3	V106A, Y181I	−5.07	15	5	47	L100, K102, K103, V179, I180, I181, V189, G190, F227, L234, H235, P236, Y318	K101 (2.97), Y188 (2.57)
RT-4	V106A, Y181C, G190S	−6.01	13	1	50	L100, K103, V179, I180, C181, W229, F227, L234, H235, P236, Y318	K101 (3.28), Y188 (2.73), S190 (3.21)
RT-5	K103N, Y188L	−5.41	13	1	50	L100, K103, V106, V179, I180, Y181, W229, L234, H235, P236, Y318	K101 (2.67), L188 (2.70)
RT-6	K103N, Y188L, G190E	−3.18	07	13	16	V108, Y183, M184, K223, L228, W29	D186 (2.80, 3.14)
RT-7	Y188L	−5.65	13	2	49, 1	L100, K103, V106, V179, I180, Y181, W229, L234, H235, P236, Y318	K101 (2.94), L188 (2.74)
RT-8	K103N, G190A	−6.13	14	1	50	L100, V106, V179, I180, Y181, A190, W229, L234, H235, P236, Y318	K101 (3.09), N103(2.95), Y188(2.78)
RT-9	K103N, Y181C, G190A	−5.92	14	1	50	L100, V106, V179, I180, C181, A190, W229, L234, H235, P236, Y318	K101 (3.10), N103 (2.97), Y188 (2.71)
RT-10	L100I, K103N, Y181C, G190A	−5.99	16	2	35, 15	I100, V106, V179, I180, Y181, V189, A190, F227, W229, L234, H235, P236, Y318	K101 (2.98), N103 (2.59), Y188 (2.68)
RT-11	K103N, Y181C	−5.72	13	1	50	L100, V106, V179, I180, C181, W229, L234, H235, P236, Y318	K101 (3.09), N103 (2.98), Y188 (2.71)
RT-12	K101E	−6.03	13	1	50	K103, V106, V179, I180, Y181, W229, L234, H235, P236, Y318	E101(3.06), Y188(2.76)

Lys103Asn is reported in literature as the most common mutation observed during therapy with first generation NNRTIs as if increases the stability of the closed form of the hydrophobic pocket [14,27,30,31,32,33]. Interestingly, this replacement did not seem to influence interactions between THD and most mutants (RT-1, RT-5, RT-6, RT-8, RT-9, RT-10 and RT-11) with the exception of RT-6. Furthermore, this mutation led to a hydrogen bond with the substituted residue at RT-1, RT-8, RT-9, RT-10 and RT-11 with distances ranging from 2.59 to 2.98 Å (Figure 4).

Leu100Ile, Val106Ala, Val108Ile, Tyr181Cys/Ile, Gly190Ala/Glu/Ser mutations are responsible for inducing steric hindrance with the NNRTIs due to the size of the side chains of these residues substituents [12,30,31,33,34]. Leu100Ile is an isosteric replacement that causes either a steric hindrance or a destabilization of the hydrogen bond with Lys101. Wang and co-workers (2001) [35] explained that, for this mutation, it is important to consider the inhibitor size and flexibility adjacent to hydrogen-bonding sites for antiviral drug design. The analysis of THD docking within RT Leu100Ile mutant (RT-10) showed that the combination with Gly190Ala influences the orientation of the diterpene in the binding site. Important interactions with residues Val189, Ala190 and Phe227 were observed in this complex. Recent SAR and molecular modeling studies performed by Das and coworkers (2012) showed some derivatives with seahorse conformation due to mutations that confer steric resistance, particularly Gly190Ala [36].

Gly190 may also be replaced by other residues (Ala/Cys/Glu/Gln/Ser/Thr or Val) leading to the same steric hindrance, but with larger influence differences on viral replication [37,38]. Docking of THD performed with RT presenting Gly190Ala substitution (RT-8, RT-9 and RT-10) showed van der Waals interaction with alanine as the main difference, due to the side chain size. In the case of RT-4 mutant (Gly190Ser) this substitution induced a small displacement of THD in the NNIBP, to a van der Waals interaction with Phe227 and a hydrogen bond with the substituted serine (d = 3.21 Å).

**Figure 5:**
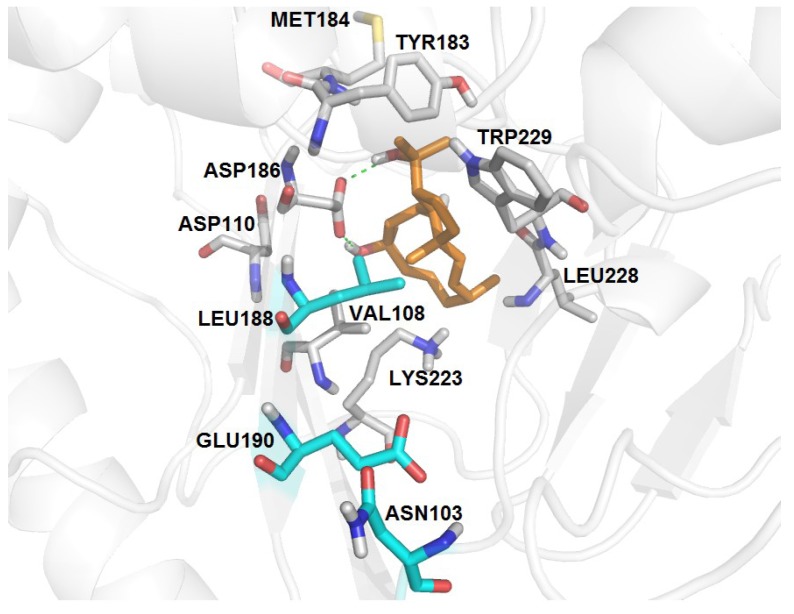
Molecular docking of THD on NNIBP of HIV-1 RT-6 mutant. Ligand is shown in orange, residues involved on the interactions between THD and the enzyme are in gray, mutated residues are highlighted in cyan and hydrogen bonds in green.

Interestingly, the Gly190Glu mutation is the substitution that mostly influences the interaction of THD with the binding site of RT-6 mutant (Lys103Asn, Tyr188Leu, Gly190Glu). The side chain of glutamic acid occupies the cavity of the binding site, leading to a steric hindrance. Although the THD is displaced from NNIBP, its inhibitory profile is maintained. Docking using this mutant resulted in 13 clusters positioning THD out of the NNIBP. Among them, we analyzed the lowest energy and the most populous clusters. The best docking result from the lowest energy cluster showed THD involved in two hydrogen bonds with Glu138, from p51 subunit, whereas the best docking result from the most populous cluster revealed two hydrogen bonds with Asp186, a residue from the recognition triad activity (Asp110, Asp185 and Asp186). Thus, despite THD being positioned outside the hydrophobic pocket due to the presence of the side chain of glutamic acid, the diterpene is located in a position that seems to block the path of the RNA or DNA primer, which may interfere in the process of reverse transcription (Figure 5).

The Tyr181Cys mutation (RT-4, RT-9, RT-10 and RT-11) did not seem to change the pattern of interactions between the THD and the enzyme when compared with docking in HIV-1 RT wild type. On the other hand, replacement by isoleucine (Tyr181Ile, RT-3), led to the loss of van der Waals interactions with Val106 and Trp229 residues, apparently substituted by new interactions with Lys102, Val189 and Phe227 residues (Figure 4). These data suggest a displacement of THD inside the NNIBP site due to isoleucine longer side chain.

Some van der Waals interactions were observed between THD and residue at position 106 of all mutants. The only exceptions are RT-3, RT-4 and RT-6 (Lys103Asn, and Tyr188Leu Gly190Glu). RT-3 and RT-4 presented the substitution of an alanine for valine, which apparently removed favorable contacts with the inhibitor due to the significant size reduction of the side chain.

Lys101Glu and Pro225His mutations are described in literature as responsible for compromising the interaction of RT with NNRTIs, due to the absence of a hydrogen bond stabilizer, (Lys101Glu), or reduction of electronic interaction and van der Waals [27,30,31,33]. Interestingly, the analysis of the molecular docking of THD with Pro225His mutant (RT-2) did not decrease the interactions between this ligand and the enzyme (Figure 4).

Due to the importance of Lys101 on the orientation of NNRTIs in the binding pocket, and the existence of a mutation at this position in HIV-1 infected patients, we performed a molecular docking in the enzyme presenting the Lys101Glu mutation, the most common according to the literature [32] (Figure 4). The evaluation of the best docking pose showed that the hydrogen bond with the residue of position 101 is maintained in the presence of mutation probably because the hydrogen bond occurs between the hydroxyl of THD and the carbonyl oxygen of the residue backbone. Therefore, the mutation of this position may not significantly affect the inhibitory activity of THD.

Interestingly, interactions with Trp229 occurred in all mutants, except for RT-3. This residue belongs to HIV-1 RT NNIBP and is described in literature as highly conserved.

Based on these data and to further enhance the affinity between the enzyme and THD expanding the action spectrum against other possible mutations, we propose the addition of an aromatic moiety in the double bond of the diterpene with the purpose of creating a π–π stacking interaction with the residue Trp229, the highly conserved enzyme HIV-1 RT. These findings could provide some insight into the THD diterpene potent activity against common NNRTI-associated resistance mutations.

## 3. Experimental Section

### 3.1. Diterpene Structures

The tridimensional structures of HDD, ADD and THD were built using the quantum chemistry software Spartan’10 (Wavefunction Inc., Irvine, CA, USA). The conformational analysis was performed using MMFF to select the lowest energy conformer for RM1 semi-empirical geometry optimization. In order to evaluate the electronic properties of diterpene compounds, they were submitted to a Single-Point *ab initio* calculation with a HF/6-31G* basis set. Thus, several electronic properties were calculated for all compounds including energies and orbital coefficients of HOMO and LUMO, HOMO-LUMO gap (E_LUMO_ − E_HOMO_), molecular dipole moment and vector.

### 3.2. Molecular Docking

In order to investigate the binding mode of HDD, ADD and THD diterpenes into RT, automated docking studies were carried out using AutoDock4.2 program running on a Windows based PC. The 3D structure of each ligand was constructed using Spartan’10 as previously described for calculating the stereoeletronic properties. The electrostatic charges were added; the structures were saved and transferred to Autodock program to create the ligand input file. The same was done with TIBO for re-docking procedure. The coordinates of wild-type HIV-1 RT crystal structure were obtained from Protein Data Bank [39] (PDB code 1HNV) [40]. RT mutants used herein correspond to those present in the experimental work carried out by Cirne-Santos and coworkers (2008) [28]. Further, we also evaluated Lys101Glu substitution, an important mutation which promotes resistance to the treatment with NNRTIs (Figure 3). To construct these RT mutants we performed amino acid substitutions in the RT wild type structure using DeepView/Swiss-PDB Viewer Version 4.0 program [41] followed by energy minimization. After modifications, the obtained models were validated by Ramachandran plot analysis using the program Procheck [42].

Docking simulations require two basic methods: a search method for exploring orientation and the conformational space available to the system and a force field to evaluate the energy of each complex. Empirical free energy force fields, which define simple functional forms for ligand–protein interactions, and semi-empirical free energy force fields, which combine traditional molecular mechanics force fields with empirical weights and/or empirical functional forms, have been used by a wide variety of computational docking methods [43].

AutoDock4 force field, which has been calibrated using a large set of diverse ligand–protein complexes, includes two major advances. The first is the use of an improved thermodynamic model of the binding process, which now allows inclusion of intramolecular terms in the estimated free energy. The second one, is that force field includes a full desolvation model that comprises terms for all atom types, including the favorable energetics of desolvating carbon atoms as well as the unfavorable energetics of desolvating polar and charged atoms. The force field also incorporates an improved model of directionality in hydrogen bonds [43].

The docking area was defined using the AutoGrid module. First, AutoGrid component pre-calculates a three-dimensional grid of interaction energies based on the macromolecular target. The cubic grid box of 60 × 60 × 60 size with spacing of 0.418 Å was generated and grid maps were created. Then automated docking studies were carried out using the empirical free energy function and the Lamarckian Genetic Algorithm (LGA) applying a standard protocol, with an initial population of 150 randomly placed individuals and a maximum number of 2.5 × 10^6^ energy evaluations, a maximum number of 27,000 generations, a mutation rate of 0.02, a crossover rate of 0.80 and an elitism value of 1. A total of 50 independent docking runs were carried out for each compound and the parameters were set using ADT (AutoDockTools). Structures differing by less than 2.0 Å in positional root-mean-square deviation (RMSD) were clustered together and the most favorable free binding energy complex structure was selected. The inhibition constants were calculated based on the dissociation of the enzyme inhibitor complex and the thermodynamics formula of ΔG = RT ln *K*_i_ [44] leading to the “Estimated Free Energy of Binding” (kcal/mol).

## 4. Conclusions

In this work we theoretically analyzed our previous reports concerning three diterpenes’ promising antiviral activity [23,26]. Overall, the SAR studies showed that a low dipole moment and high HOMO-LUMO gap values are related to the antiviral activity. All diterpenes showed low theoretical toxicity, similar to previous *in vitro* experiments as well as good oral bioavaliability. According to our molecular docking results with RT wild type, HDD and ADD presented a conformation similar to first generation NNRTIs. On the other hand, THD showed a seahorse-like conformation, which is maintained within all complexes, except for RT-6 mutant. Interestingly, the hydrogen bond with the residue Lys101 is also preserved even with the substitution of this amino acid. Finally, we proposed a new derivative with an aromatic moiety in the double bond of the diterpene to increase the affinity for the target through a π–π stacking interaction with residue Trp229, highly conserved in the enzyme. These findings could provide some insight into the THD diterpene potent activity against common NNRTI-associated resistance mutations.
